# COVID-19: Unveiling the Role of Uncertainty in Medical Education

**DOI:** 10.15694/mep.2020.000166.1

**Published:** 2020-08-12

**Authors:** Lindsay Herzog

**Affiliations:** 1 University of Toronto

**Keywords:** Medical education, COVID-19, uncertainty, clinical reasoning

## Abstract

This article was migrated. The article was marked as recommended.

Since the start of COVID-19, we have all heard the phrase “facing unprecedented uncertainty,” and it is this uncertainty that health care professionals are navigating on a daily basis. In this personal view article, I highlight the impact of uncertainty on everyday clinical practice, and the amplification of this during the current pandemic. In light of this, I argue for the value of teaching for uncertainty in medical education in a way that is transparent and intentional. Ultimately, I query whether such a curricular change might allow trainees, health care professionals and the public to feel more prepared when facing these uncertain times.

## Article

As a medical educator, I think often about the roots of my education - about the way in which I was taught and the ways in which I hope to teach. I think about mentors I’ve had who have pushed me to think about my education in an effort to motivate me to educate future generations of medical trainees in a way that is transformational, critical, and intentional. I also think about the heavy reliance on evidence-based medicine that pervaded my medical training - the guidelines that I absorbed in order to feel well-prepared for my licensing exam, with the ultimate hope of feeling well-prepared for stepping out into my first year of practice of family medicine. I was warned that I wouldn’t feel well-prepared, and that it takes at least five to seven years to feel comfortable more times than not. But I was reminded that I could always go back to the guidelines; I could always look up my question or ask a colleague. The Internet would become my best friend and I could always look to it in times of uncertainty.

This was a very accurate image of my first six months of family medicine practice. Many clinical visits ended with “Okay, I’m going to look into this more and I’ll get back to you with thoughts on possible next steps.” I would spend subsequent hours researching my clinical questions, seeking the input of my colleagues and consultants. I would do this until I felt confident that I was offering the very best suggestions to allow the patient to make an informed decision regarding their care. Yet despite all of this, I was still left with a sense of unease. I still didn’t know what the outcome would be of the plan we put in place. Would the patient follow-up if things worsened, as we had discussed? Would they know how to seek the care they need? Was I even right about the diagnosis in the first place?

I faced this same discomfort in my undergraduate medical training, and I recognized that very rarely was there ever a single “right” answer in medicine. This became increasingly apparent in my family medicine residency, where ambiguity and vague clinical presentations were pervasive. Though the conversation about uncertainty was more transparent in my residency training, I owe this to the specific mentors and teachers I had. There was no formal curriculum on uncertainty, and having never heard this kind of dialogue in the four previous years of medical school, it came as a shock. Why was this not woven throughout my training, alongside evidence-based medicine? It felt to me that medical education was trying to eliminate uncertainty, instead of revealing its value. We have guidelines and evidence-based medicine to look to. We devalue gut feelings, expert opinion, and personal experience. These players sit on the sidelines while the evidence takes the spotlight. And though there is substantial rationale for the existence and need for evidence, trainees learn to see everything else as “fluff” and “soft skills.” Consequently, the public sees doctors as those who always have the right answers and in turn, training physicians learn to view themselves in this way. We don’t spread the message that we are oftentimes unsure of the cause of their rash, their fatigue, the tingling sensation in their left index finger. We don’t spread the message that this uncertainty keeps us up at night.

While this concept of uncertainty had been on my mind since starting in practice, its relevance to medical practice was recently brought to light in a way I never could have anticipated.

Cue December 2019: We begin to hear rumblings of the novel coronavirus. It sounds remote and somewhat irrelevant.

January 2020: We begin screening patients for recent travel from the Wuhan region. We’re reassured “the risk to Canadians remains low.”

February 2020: The virus appears to be spreading. Greater precautions are being taken “out of an abundance of caution, though we want to reassure everyone that the risk to Canadians remains low.”

March 2020: I see a patient who had recently traveled to California. She has a very mild intermittent cough that she didn’t feel was necessary to report at the front screening station. Besides, the U.S. had only recently joined the list of countries where the infection seems to be spreading. I wonder if I’ve been exposed. The public health hotline is becoming overwhelmed and there’s no answer after hours on hold. The infection control specialist at the hospital where I work is unable to provide guidance for employees, only for patients. Occupational Health states I am safe to be at work, and if I develop symptoms to call them back. Does my irritated throat after hours of talking to patients count as a symptom? Could it be COVID? Am I unknowingly infecting my colleagues, my patients, my partner? And so, the uncertainty began to build.

March 11 2020: WHO declares the novel coronavirus a pandemic

March 12 2020: Ontario schools close

March 23 2020: Toronto declares a state of emergency

We’re in it now.

Since this date, the rapidity of incoming information has flowed endlessly. Changing “guidelines,” new protocols, revisions to those protocols, revisions to revisions of those protocols. Processes which are different depending which clinic you’re in, which district, which province, which country. We were all making very big decisions with very little information.

Where was my lecture on preparing for a pandemic? Where was the evidence to navigate extreme PPE shortages; the existential crises that my patients were facing; the fear of my colleagues that they were a vector carrying this deadly virus between their patients and their families? Where were my guidelines when I needed them most? One thing remained constant amidst all of this: global uncertainty. Uncertainty was at an all-time high. Not just for a new-to-practice family doctor, but also for Canada’s Chief Public Health Officer, and for every other medical professional in the entire world. We were all facing unprecedented uncertainty.

In times like this, I can look back to those moments of uncertainty which were embraced by my supervisors in my residency. I can look to the ways I learned, by reading between the lines of varied opinions on a single case, that uncertainty was the rule, and not the exception **.** I can look to the fact that I have recently developed a sense of kinship with uncertainty, in my first six months of practice, knowing that this is a feeling I will continue to hold for the duration of my career. But it does make me wonder - if we overtly embraced uncertainty in medical education in a way that was intentional and transparent, might healthcare professionals be more comfortable with the current state of ever-changing protocols and “guidelines”? Might we be more accepting of the constantly conflicting studies being produced at lightning speed? Might the public continue to trust the opinions of experts when they change their advice?

Although reflection-based courses create space for dialogue in an attempt to navigate “the swampy lowlands of indeterminate practice” ( Schön, 1983), these initiatives are often isolated and disparate from the rest of medical training. This spatial separation consequently amplifies a detachment between reflecting upon uncertainty in a classroom, and evidence-based medicine in the clinical space. Alternatively, weaving these two concepts together offers potential for recognizing their collective value. For example, when reviewing a patient case, we might consider encouraging a learner to think about the role of customizing a guideline to meet their unique individual needs, while creating a dialogue about the uncertainty that is experienced when doing this. Tonelli and Upshur (2019) have advocated for teaching for uncertainty through a philosophical lens, and embedding it within existing curricular activities such as during bedside teaching and evidence-based medicine modules. An argument also exists for the integration of uncertainty into clinical reasoning assessment methods, further substantiating the role it plays in everyday clinical practice ( Cooke and Lemay, 2017).

While medical education may not be able to alter the trajectory of a pandemic, perhaps an integrated approach to the inclusion of navigating uncertainty in medical training would prepare learners and healthcare professionals to face the unknown. As we move through COVID-19, can we begin to see the value in teaching for uncertainty?

## Take Home Messages

• The COVID-19 pandemic has highlighted the potential value of teaching for uncertainty in medical education
• The concept of teaching for uncertainty in a way that is transparent and integrated must be recognized beyond the current context in order to facilitate trainees in navigating the unknown in their everyday clinical practice

## Notes On Contributors

**Lindsay Herzog,** MD CCFP is lecturer, Mount Sinai Hospital, Department of Family and Community Medicine, University of Toronto, Toronto, Ontario, Canada.
