# Supplementary material for: COVID-19: Unveiling the Role of Uncertainty in Medical Education
Source: MedEdPublish (2016). 2020 Aug 12;9:166. [Version 1] doi: 10.15694/mep.2020.000166.1 (PMC10702646; doi:10.15694/mep.2020.000166.1)
Supplement: Peer review history

# Reviewer report: Reviewer response for version 1

**DOI:** 10.21956/mep.20096.r30964

**Published:** 2020-08-27

**Authors:** Johnny Lyon Maris

**Affiliations:** 1 Southampton GP Education Unit

This review has been migrated. The reviewer awarded 4 stars out of 5 As a piece of narrative from a Family Physician this article talks about what we do on a daily basis in Primary Care. We are presented with a bundle of symptoms, make quick decisions on whether they are part of an established pattern, use time and investigations as diagnostic tools and live with uncertainty. Our curriculum have a large section on dealing with risk and living with uncertainty as I am sure very Family Medicine curriculum has across the world. The uncertainty here in COVID-19 is not the diagnosis but the outcome, as the disease follows no patterns. Apparently well people can rapidly deteriorate, and the course is unpredictable. So as primary care physicians we are not dealing with the uncertainty of diagnosis but of the outcome and that is new in our specialty.This article highlights the importance of dealing with uncertainty and uses the narrative form well. Thank you for submitting.

Reviewer Expertise:

NA

No decision status is available

# Reviewer report: Reviewer response for version 1

**DOI:** 10.21956/mep.20096.r30967

**Published:** 2020-08-18

**Authors:** P Ravi Shankar

**Affiliations:** 1 American International Medical University

This review has been migrated. The reviewer awarded 4 stars out of 5 I enjoyed reading this brief article which may have profound lessons for medicine and medical education. Medicine has been founded on a positivist quantitative paradigm. I did not come across much of an understanding about and acceptance of uncertainty during my undergraduate medical course. I believe slowly medicine has become more tolerant of uncertainty and of qualitative research and does not consider statistics and quantitative research as the only possible options. The pandemic has caused some fundamental uncertainty. Most people now work from home blurring the boundaries between the work and the personal space. Most learning and even consultations are now happening in the virtual space. We regard evidence-based medicine and randomized clinical trials as the gold standard. This has served us well for most diseases and conditions. Over the years many authorities have pointed out many inconsistencies in clinical trials. These trials are mostly conducted by the pharmaceutical companies which have discovered or produced the drug they are testing. The motivation for profit is a predominant driving force. We mainly compare the new drug against a sugar pill and sometimes against existing drugs. For COVID-19 where there are no existing drugs many study designs compare different candidate drugs or a candidate drug against standard care. What weightage should we give to the impressions of many clinicians who strongly believe that a particular drug is showing promising effects. Uncertainty may be the only constant associated with this pandemic during these times of conflicting results and rapidly changing parameters.

Reviewer Expertise:

NA

No decision status is available

# Reviewer report: Reviewer response for version 1

**DOI:** 10.21956/mep.20096.r30963

**Published:** 2020-08-17

**Authors:** Thomas Puthiaparampil

**Affiliations:** 1 UNIVERSITI MALAYSIA SARAWAK

This review has been migrated. The reviewer awarded 3 stars out of 5 As a medical educationist for the past 2 decades, I haven't come across any special course on preparedness for a pandemic. Our focus is on the basics of human anatomy, physiology, microbiology, biochemistry, pharmacology, etc. apart from clinical subjects. Medical emergencies are given lots of importance in the curriculum. Students are exposed to acute presentations and their management. Pandemics being rare, it is not possible to make it a course in itself. In my opinion, COVID-19 caught the health profession as well as most governments of the countries unprepared. However, most countries took appropriate steps to contain the disease and minimize mortality, although many countries failed miserably. All these reveal the prevailing uncertainty. It is not practically possible to be ready for such rare and uncertain situations all the time. Situations have to be tackled as they appear. For medical students, if they are well trained in the basics and how to approach and handle emergencies, that will be good enough. Highlighting too much of rarities is likely to divert the attention and focus away from the more common issues.

Reviewer Expertise:

NA

No decision status is available

# Reviewer report: Reviewer response for version 1

**DOI:** 10.21956/mep.20096.r30966

**Published:** 2020-08-13

**Authors:** Ben Canny

**Affiliations:** 1 Monash University

This review has been migrated. The reviewer awarded 5 stars out of 5 This is a very interesting piece highlighting a critical issue in education and health professions practice. Dealing with ambiguity and uncertainty is a hallmark of the human condition, and needs to to developed and nurtured among health care professions. Ironically, a formal consideration of the scientific method also champions uncertainty (Popper/falsificationism), and a well trained health professional, even if they imagine themselves as a scientist, should embrace uncertainty. Of interest, if one is to read the history of Bayes' theorem, which relies upon making a stab at a prior probability, and then improving it with new data, the frequentists (eg Fisher) objected to it as they didn't like the idea of "making a guess". Herzog has opened us to some important principles and ideas.

Reviewer Expertise:

NA

No decision status is available

# Reviewer report: Reviewer response for version 1

**DOI:** 10.21956/mep.20096.r30965

**Published:** 2020-08-12

**Authors:** Virginia Randall

**Affiliations:** 1 Uniformed Services University of the Health Sciences

This review has been migrated. The reviewer awarded 5 stars out of 5 This is a most impressive summary of the conundrum that is at the core of clinical medicine. Our own work on students' reaction to confronting uncertainty was that it was a surprise disillusionment. They weren't supported as the authors suggest, and often the disillusionment lead to doubting themselves and fearing the future.

Reviewer Expertise:

NA

No decision status is available
